# The inhibition of PLCγ1 protects chondrocytes against osteoarthritis, implicating its binding to Akt

**DOI:** 10.18632/oncotarget.23286

**Published:** 2017-12-15

**Authors:** Heguo Cai, Ning Qu, Xiaolei Chen, Yang Zhou, Xinpeng Zheng, Bing Zhang, Chun Xia

**Affiliations:** ^1^ Zhongshan Hospital, Xiamen University, Fujian 361004, China; ^2^ School of Medicine, Xiamen University, Fujian 361102, China; ^3^ The Third Hospital of Xiamen, Fujian, China, Fujian 361000, China

**Keywords:** PLCγ1, Akt, chondroprotection, rat OA model, OA chondrocytes

## Abstract

Previous studies have addressed the involvement of phosphoinositide-specifc phospholipase γ1 (PLCγ1) and protein kinase B (PKB/Akt) in osteoarthritis (OA) pathogenesis, but it is not ascertained the possibility of them to be potential targets for OA therapy. Here, through local intra-articular injection of PLCγ or Akt inhibitor in a rat OA model induced by anterior cruciate ligament transaction plus medial meniscus resection, the architecture of chondrocyte and matrix organization of articular cartilage were observed using histopathological assays and Aggrecan, Col2, PLCγ1, and Akt levels were detected using immunohistochemistry assays. By treatment of Akt or PLCγ inhibitor and transfection of different PLCγ1- or Akt-expressing vectors in rat OA model chondrocytes, Aggrecan, Col2, PLCγ1, p-PLCγ1, Akt, and p-Akt levels were detected using western blotting analysis. The binding between PLCγ1 and Akt was assessed with co-immunoprecipitation assays in human OA chondrocytes. These results showed that PLCγ inhibition protected chondrocytes against OA, but Akt inhibition did not dramatically aggravate OA progression. There were mutual antagonism and binding between PLCγ1 and Akt that could be regulated by their phosphorylation levels. Consequently, the data reveal that the inhibition of PLCγ1 may provide an attractive therapeutic target for OA therapy, implicating its binding to Akt.

## INTRODUCTION

Osteoarthritis (OA) is a chronic and degenerative disease of articular cartilage, mainly characterized by chondrocyte death and articular cartilage matrix degradation in OA pathogenesis. Thus, either suppressing matrix degradation or promoting matrix synthesis could mitigate or prevent OA degeneration. Some signal molecules, such as p38, JNK and ERK MAP kinases, and PI-3 kinase/Akt, could regulate the expression levels of Aggrecan(a proteoglycan) and type II collagen (Col 2), which are main components in articular cartilage matrix, to suppress or promote matrix synthesis [1]. Recent studies suggest targeting some signaling molecules in treatment of OA, because it is more effective than current treatments and less harmful to normal cells. Lentiviral vector-mediated shRNAs targeting a functional isoform of the leptin receptor (Ob-Rb) inhibit cartilage degeneration in a rat model of osteoarthritis [2]. Local intra-articular injection of rapamycin(mTOR inhibitor) or cartilage-specific deletion of mTOR can delay articular cartilage degeneration in a murine model of OA or protect mice from osteoarthritis [3, 4]. Targeting the damage-associated molecular patterns (DAMPs) may be the potential therapeutic strategies for the treatment of OA [5]. Therefore, targeting the signal molecules involved in chondrocyte metabolism may be a potent solution for OA therapy.

Phosphoinositide-specifc phospholipase γ (PLCγ), including PLCγ1 and PLCγ2, could be activated by many extracellular factors to induce hydrolysis of phosphatidylinositol 4,5-bisphosphate(PtdIns(4,5)P2) to form the second messages diacylglycerol(DAG) and inositol 1,4,5-trisphosphate(IP3). Both DAG and IP3 in turn activate a number of signaling pathways to regulate cell metabolism in mammalian cells [6]. PLCγ1 is expressed in most mammalian cells, while PLCγ2 is mainly expressed in immune cells. Thus, most research focuses on PLCγ1. Some authors’ studies have already addressed the expression pattern of PLCγ in chondrocytes. PLCγ is associated with the sex-specific response of rat costochondral cartilage growth plate chondrocytes to 17β-estradiol [7]. Periodic mechanical stress not only causes chondrocyte area expansion and migration, but also activates PLCγ1/Y783 [8]. Moreover, PLCγ1 is implicated in some important signaling pathways to regulate chondrocyte processess. PLCγ mediated FGFR3-induced STAT1 activation and this signaling cascade involved in the induction of apoptosis in the chondrogenic cell line ATDC5 [9]. The Src-PLCγ1-ERK1/2 signaling transduction pathway is involved in cartilage tissue integration by affecting chondrocyte migration [10]. Periodic mechanical stress also promotes chondrocyte proliferation and matrix synthesis in part through the Src-PLCγ1-MEK1/2-ERK1/2 signaling pathway [11]. PDGF was able to activate the GIT1- and PLCγ1-mediated ERK1/2 pathway to control chondrocyte proliferation [12]. However, the role of PLCγ1 in OA remains clear. Our recent observation also showed that PLCγ1 was expressed at elevated level in human OA chondrocytes than in normal chondrocytes, serving as a pivotal element of signal cascades constituted by ERK and mTOR to promote matrix degradation in human OA chondrocytes [13, 14]. We then hypothesized that PLCγ1 could be involved in OA chondrocyte metabolism, contributing to matrix degradation. In addition to PLCγ1, protein kinase B (PKB/Akt), a crucial regulator in cell proliferation and apoptosis, has been implicated in chondrocyte processes. For example, Akt has a higher expression in normal chondrocytes than in OA chondrocytes, required for both basal and insulin-induced type II collagen (Col 2) expression in chondrocytes [15]. The expression of the Akt inhibitor called TRB3 (tribbles homolog, a pseudokinase that contains a truncated kinase domain and binds to Akt to inhibit Akt phosphorylation and activation) increases in human OA cartilage, and inhibits insulin-like growth factor 1-mediated cell survival and proteoglycan synthesis [16]. We previously show that the activation of Akt contributed to the chondroprotection of nicotine, berberine, and morroniside on OA chondrocytes through promoting matrix synthesis, respectively [17–19]. Therefore, the increased Akt has a potent chondroprotection in OA pathogenesis. However, up to date, it is not ascertained the possibility of PLCγ1 or Akt as a potential target for OA therapy.

Although most signal molecules have been examined in isolation, it is becoming clear that there is a significant crosstalk among them and the overall effects on OA pathogenesis depend on the balance in activity of them [1]. Investigating the crosstalk among these signal molecules is indispensable to elucidate the regulatory mechanism of OA pathogenesis. It has indicated that PLCγ activation is partially dependent on the PI(3,4,5)P3 production by activated PI3K [20], the latter could simultaneously phosphorylate Akt1/2 at Ser473/474 [21], indicating that PLCγ and Akt share common upstreamer, PI3K. Hence, it is becoming clear that these two pathways are interconnected at several levels. Moreover, it has been reported that Akt interaction with PLCγ regulates the G(2)/M transition triggered by FGF receptors from MDA-MB-231 breast cancer cells [22]. Our previous study indicated that the interaction between PLCγ and Akt was involved in cell metastasis in human gastric adenocarcinoma [23]. Therefore, we hypothesize that there exists a crosstalk between PLCγ and Akt to be involved in OA pathogenesis.

In this study, it is investigated the role of PLCγ and Akt and the relationship between them in OA chondrocytes. We showed that local intra-articular injection of PLCγ inhibitor could protect chondrocytes against OA in a rat OA model, whereas Akt inhibitor could not dramatically aggravate OA progression. There were mutual antagonism and binding between PLCγ1 and Akt that could be regulated by their phosphorylation levels. Therefore, the inhibition of PLCγ1 may serve as a better solution for OA therapy than Akt activation, partially, implicating its binding to Akt.

## RESULTS

### Effect of PLCγ1 and Akt inhibitors on articular cartilage in a rat OA model

**Figure 1:**
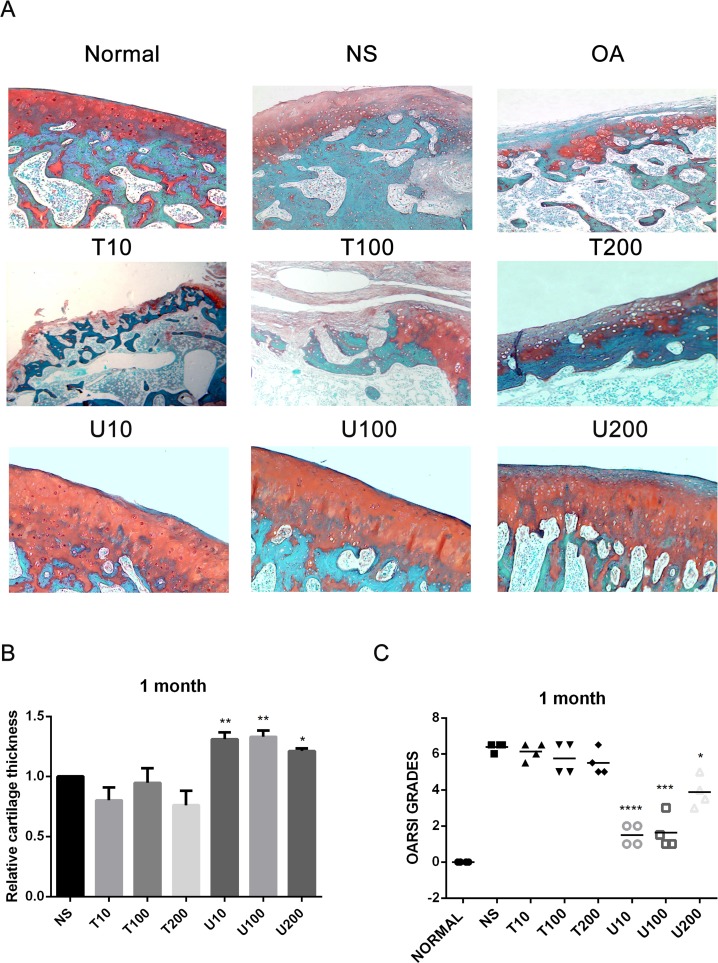
Histopathological evaluation of OA in a rat OA model treated with different inhibitors of PLCγ1 and Akt for 1 month (**A**) Representatives images of Safranin O –Fast green staining from the rats treated by different inhibitors of PLCγ1 and Akt after ACLT+MMx (original magnification ×100). (**B**) Graph indicating the relative cartilage thickness of each femur condyle (from superficial zone to tidemark). (**C**) Graph indicating the OARSI scores (^*^P < 0.05, ^**^P < 0.01, ^***^P < 0.001, ^****^P < 0.0001, *vs* NS group).

**Figure 2:**
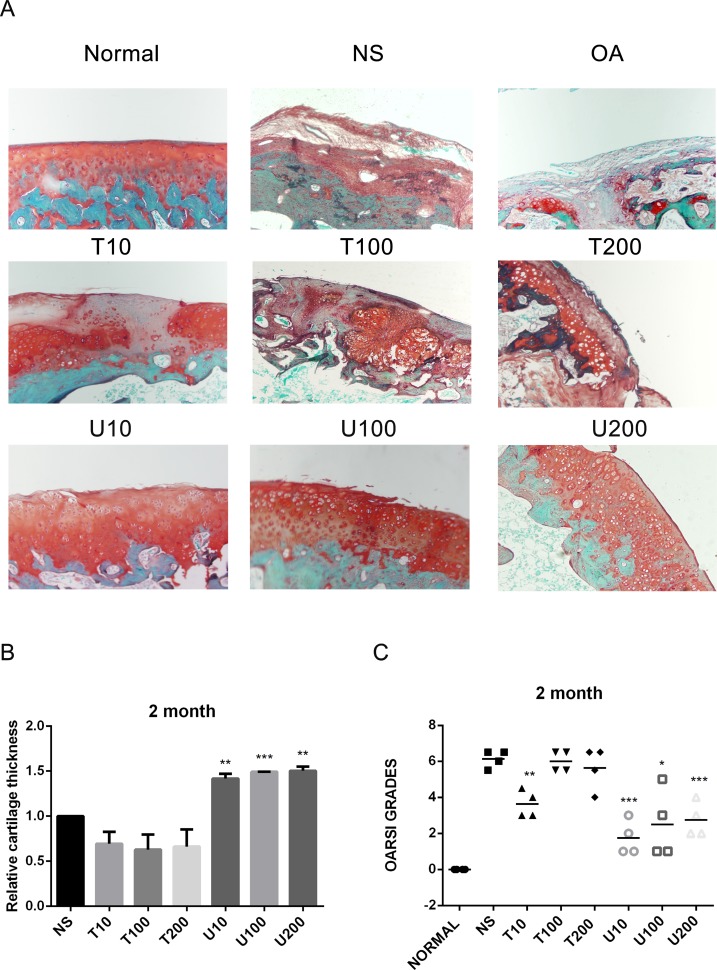
Histopathological evaluation of OA in a rat OA model treated with different inhibitors of PLCγ1 and Akt for 2 month (**A**) Representatives images of Safranin O –Fast green staining from the rats treated by different inhibitors of PLCγ1 and Akt after ACLT+MMx (original magnification ×100). (**B**) Graph indicating the relative cartilage thickness of each femur condyle (from superficial zone to tidemark). (**C**) Graph indicating the OARSI scores (^*^P < 0.05, ^**^P < 0.01, ^****^P < 0.0001, *vs* NS group).

Given the number of signal cascades triggered by activated Akt, which might make it difficult to understand its regulatory mechanism, the effect of Akt on articular cartilage was detected with the treatment of its pharmaceutical inhibitor Triciribine (TCN) in this study. Using a rat OA model induced by ACLT+MMx, we investigated the effect of PLCγ1 and Akt inhibition on articular cartilage with local intra-articular injection of its pharmaceutical inhibitor U73122 (U) and TCN. Rats were killed after 1 and 2 month-injection with U73122 or TCN, respectively, the specimens were then fixed, sectioned into 3 μm thicknesses, and stained with H.E. and Safranin O –Fast green stainings. The results after injection for 1 and 2 month were shown in Figures 1 and 2, respectively. Figures 1A and 2A displayed the smooth cartilage surface of femur condyle, normal architecture of matrix, and intact and appropriate cell distribution in normal group, and the heavy degeneration of articular cartilage in both OA and NS (OA group intra-articularly injected by 0.9% normal saline) groups. Meanwhile, in 1 month-treated groups, the injection of U73122 at different concentrations enhanced the thickness of articular cartilage in femur condyle and number of chondrocytes, and reducing the grade of Osteoarthritis Research Society International (OARSI) scoring system, in which 10 μg/mg U73122 was most effective, indicating the protective effect of U73122 on articular cartilage (Figure 1, ^*^P < 0.05, ^**^P < 0.01, ^***^P < 0.001, ^****^P < 0.0001, *vs* NS group). Nevertheless, the architecture of chondrocyte and matrix of articular cartilage in TCN-treated groups at different concentrations were similar to that in NS group along with the decreased thickness of articular cartilage and increased OARSI scores (Figure 1). Like 1 month-treated groups, the protective effect of U73122 on OA articular cartilage was observed in 2 month-treated groups (Figure 2, ^*^P < 0.05, ^**^P < 0.01, ^***^P < 0.001, ^****^P < 0.0001, *vs* NS group). Although TCN (10 μg/kg) caused a significant decrease at the grade of OARSI scoring system compared with NS group (Figure 2C, ^**^P < 0.01), the other two concentration of TCN did not change the thickness of articular cartilage and OARSI scores compared with NS group. Taken the data together, U73122 treatment for either 1 or 2 month had a significant chondroprotective effect on articular cartilage in a rat OA model, but TCN had not any intensifying effect on OA cartilage degeneration.

### Effect of PLCγ1 inhibitor on the expression levels of Aggrecan and Col 2 in a rat OA model

**Figure 3:**
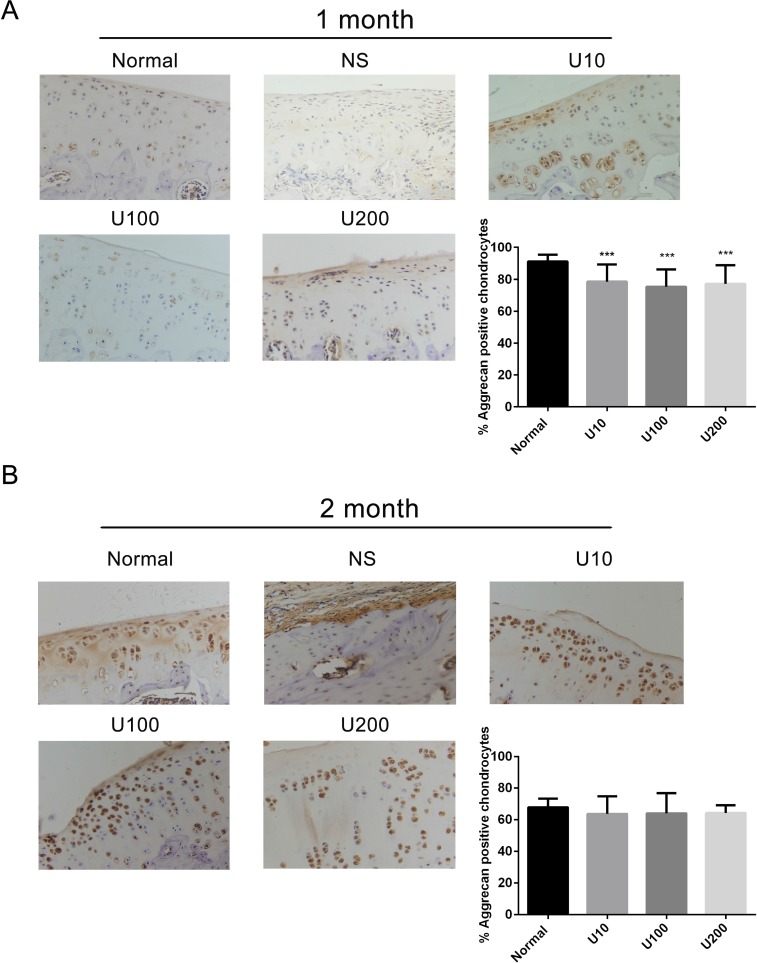
Effect of PLCγ1 inhibitor on Aggrecan expression in a rat OA model Specimens were longitudinally cut into 3 μm sections and the levels of Aggrecan expression level were detected by immunohistochemisty technique (original magnification ×200). As described in Material and Methods, the positive chondrocytes were counted and analyzed using Image-Pro Plus 6.0 Software and GraphPad Prism version 5. (**A**) Representative images from rats treated by PLCγ1 inhibitor for 1 month after ACLT+MMx (original magnification ×200). Graph indicating the percentage of positive chondrocytes expressing Aggrecan (^***^P < 0.001, *vs* normal group). (**B**) Representative images from rats treated by PLCγ1 inhibitor for 2 month after ACLT+MMx (original magnification ×200). Graph indicating the percentage of positive chondrocytes expressing Aggrecan.

**Figure 4:**
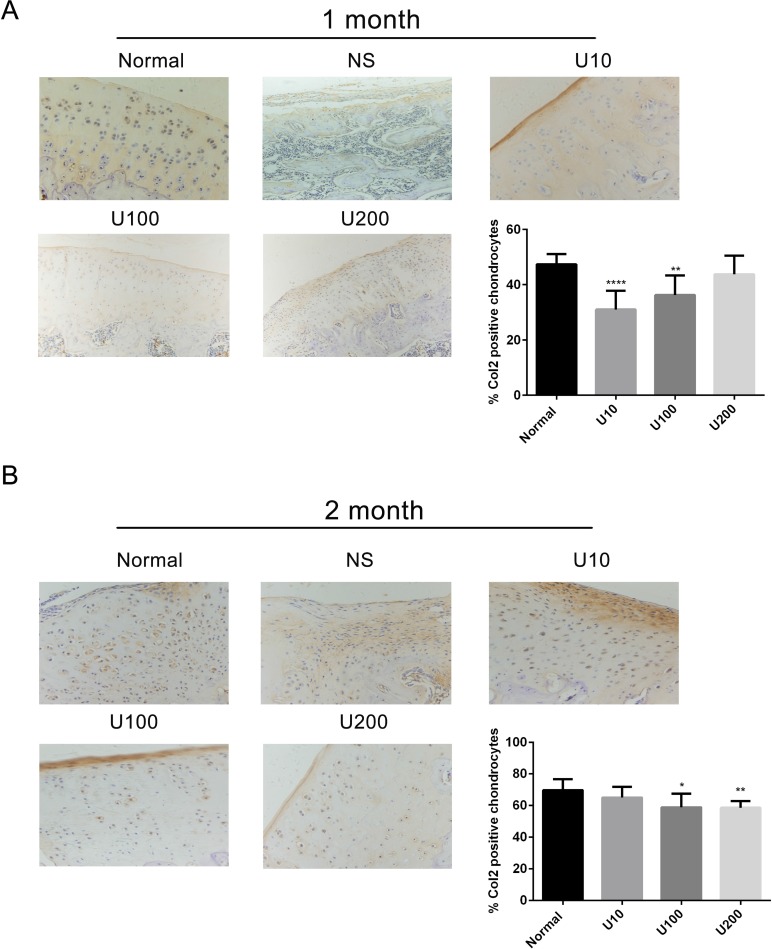
Effect of PLCγ1 inhibitor on Col 2 expression in a rat OA model Specimens were longitudinally cut into 3 μm sections and the levels of Aggrecan expression level were detected by immunohistochemisty technique (original magnification ×200). As described in Material and Methods, the positive chondrocytes were counted and analyzed using Image-Pro Plus 6.0 Software and GraphPad Prism version 5. (**A**) Representative images from rats treated by PLCγ1 inhibitor for 1 month after ACLT+MMx (original magnification ×200). Graph indicating the percentage of positive chondrocytes expressing Col2 (^**^P < 0.01,^****^P < 0.0001, *vs* normal group). (**B**) Representative images from rats treated by PLCγ1 inhibitor for 2 month after ACLT+MMx (original magnification ×200). Graph indicating the percentage of positive chondrocytes expressing Col 2 (^*^P < 0.05, ^**^P < 0.01, *vs* normal group).

Aggrecan and Collagen Type 2(Col 2) are main biomarkers of cartilage matrix synthesis, the effect of PLCγ1 inhibition on them was then assessed with immunohistochemistry assay in a rat OA model. Considering the heavy cartilage degeneration in NS group, the difference between U73122-treated and normal groups was analysed. In 1 month-treated group, Figure 3A showed that the expression level of Aggrecan in U73122- treated groups at different concentrations was less than that in normal group (^***^P < 0.001). In 2 month-treated groups, the expression level of Aggrecan in U73122-treated groups at different concentrations was close to that in normal group (Figure 3B). These results indicated that the treatment of U73122 for 2 month enhanced the expression level of Aggrecan, reaching normal level, and that the efficacy of U73122 treatment for 1 month was inferior to that for 2 month. In addition, the expression level of Col 2 in U73122 (200 μg/kg)-treated group at for 1 month was close to that in normal group, whereas Col 2 expressions in the other two concentrations of U73122 were significantly less than that in normal group, indicating that the efficacy of U73122 (200 μg/kg) was superior to that of the other two groups (Figure 4A, ^**^P < 0.01, ^****^P < 0.0001). In 2 month-treated groups, the expression level of Col 2 in U73122 (10 μg/kg)-treated group was close to that in normal group, whereas Col 2 expressions in the other two groups of U73122 were less than that in normal group, indicating that the efficacy of U73122 (10 μg/kg) treatment was superior to that of the two other groups (Figure 4B, ^*^P < 0.05,^**^P < 0.01). Therefore, treatment of both U73122 (200 μg/kg, 1 month) and U73122 (10 μg/kg, 2 month) could enhance the expression of Col 2 reaching normal level, and that the efficacy of U73122 may partially depend on the time and concentration of its treatment. Consequently, definite concentration PLCγ1 inhibitors promoted matrix synthesis through increasing Aggrecan and Col 2 expression levels in a rat OA model.

### Relationship between PLCγ1 and Akt in OA chondrocytes

**Figure 5:**
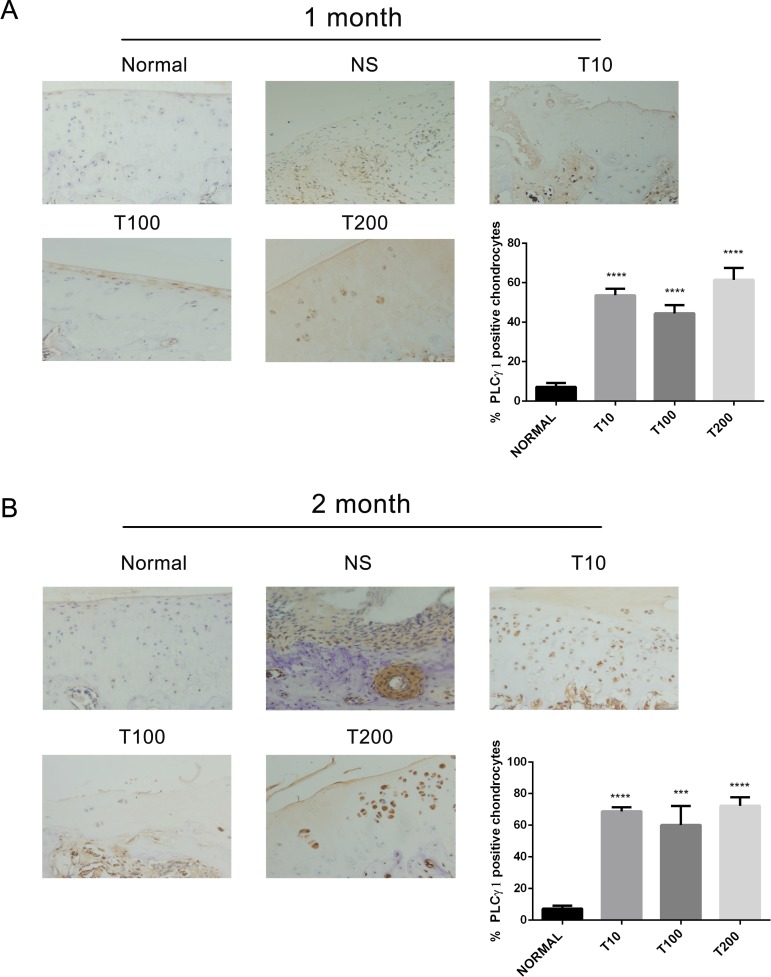
Effect of Akt inhibitor on PLCγ1 expression in a rat OA model Specimens were longitudinally cut into 3 μm sections and the levels of Aggrecan expression level were detected by immunohistochemisty technique (original magnification ×200). As described in Material and Methods, the positive chondrocytes were counted and analyzed using Image-Pro Plus 6.0 Software and GraphPad Prism version 5. (**A**) Representative images from rats treated by Akt inhibitor for 1 month after ACLT+MMx (original magnification ×200). Graph indicating the percentage of positive chondrocytes expressing PLCγ1 (^****^P < 0.0001, *vs* normal group). (**B**) Representative images from rats treated by Akt inhibitor for 2 month after ACLT+MMx (original magnification ×200). Graph indicating the percentage of positive chondrocytes expressing PLCγ1 (^***^P < 0.001, ^****^P < 0.0001, *vs* normal group).

**Figure 6:**
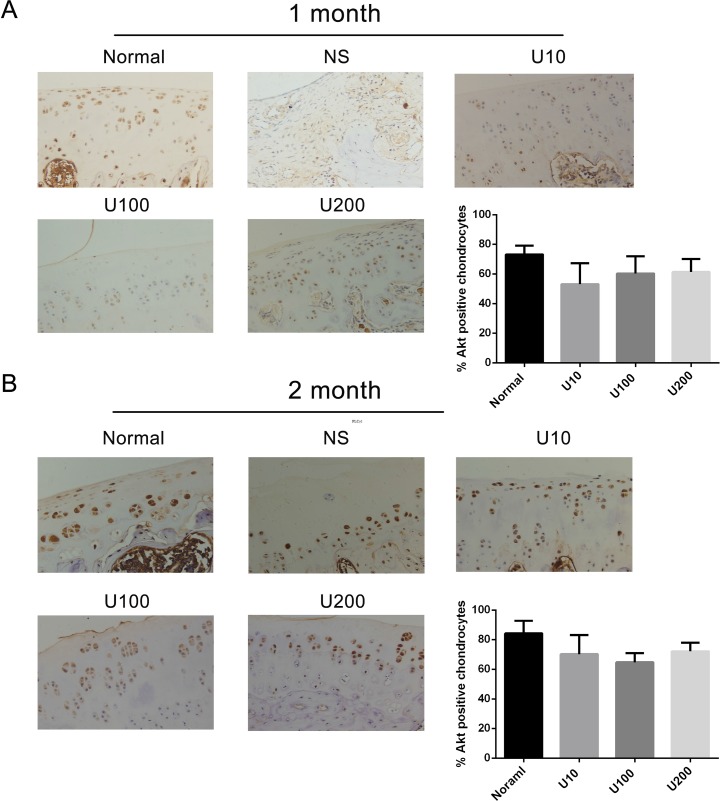
Effect of PLCγ1 inhibitor on Akt expression in a rat OA model Specimens were longitudinally cut into 3 μm sections and the levels of Aggrecan expression level were detected by immunohistochemisty technique (original magnification ×200). As described in Material and Methods, the positive chondrocytes were counted and analyzed using Image-Pro Plus 6.0 Software and GraphPad Prism version 5. (**A**) Representative images from rats treated by PLCγ1 inhibitor for 1 month after ACLT+MMx (original magnification ×200). Graph indicating the percentage of positive chondrocytes expressing Akt. (**B**) Representative images from rats treated by PLCγ1 inhibitor for 2 month after ACLT+MMx (original magnification ×200). Graph indicating the percentage of positive chondrocytes expressing Akt.

To ascertain whether there is a crosstalk between PLCγ1 and Akt in OA pathogenesis, the expression levels of PLCγ1 or Akt were assessed with immunohistochemistry assay in PLCγ1 and Akt inhibitor-treated groups, respectively. Either 1 or 2 month-treated groups, PLCγ1 had significantly higher expression in different concentration TCN-treated groups than that in normal group, indicating that TCN treatment enhanced PLCγ1 expression level (Figure 5, ^***^P < 0.001, ^****^P < 0.0001). Meanwhile, the treatment U73122 at different concentrations for either 1 or 2 month enhanced Akt expression level, close to that in normal group, indicating that U73122 also promoted Akt expression reaching normal level. Therefore, there was a reciprocal modulation between PLCγ1 and Akt, a possible mutual antagonism in chondrocytes of a rat OA model. Moreover, compared with Figures 5 and 6, the effect of U73122 on Akt expression seemed to be superior to that of TCN on PLCγ1 expression.

**Figure 7:**
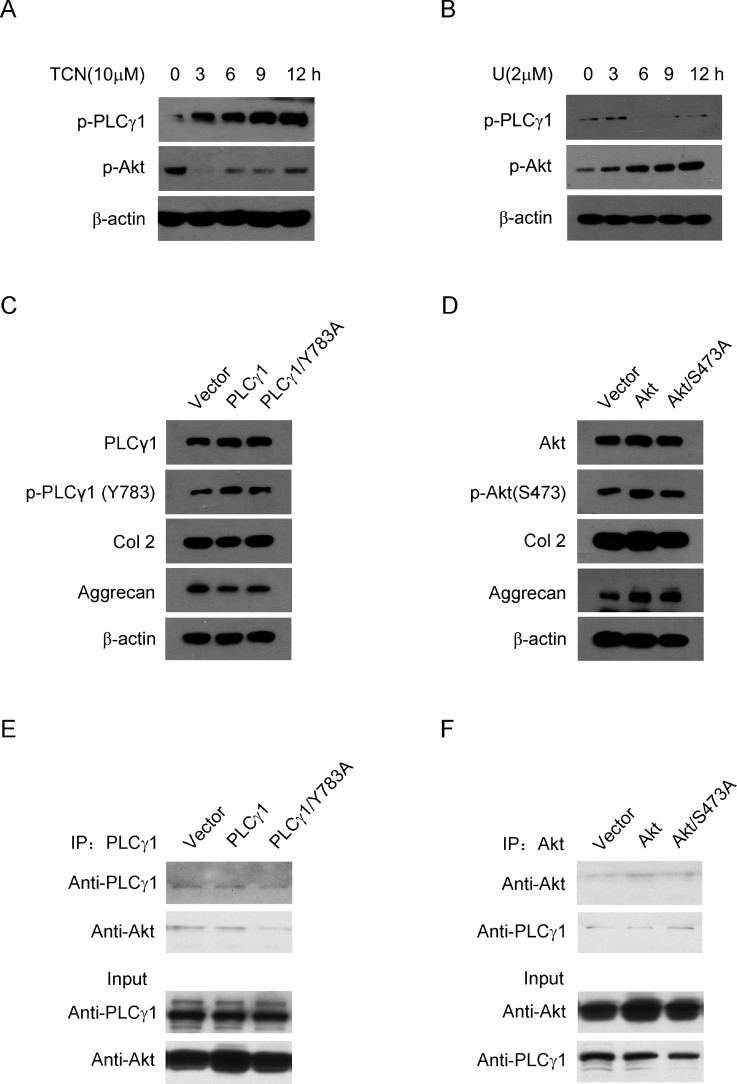
Relationship between PLCγ1 and Akt in OA chondrocytes (**A** and **B**) Rat OA model chondrocytes by IL-1β pre-treatment were treated with U73122 (2 μM) and TCN (10 μM) for 3, 6, 9, 12 h, respectively. P-PLCγ1, p-Akt, and β-actin levels were detected by western blotting analysis using anti-p-PLCγ1, p-Akt, and β-actin antibodies. (**C**) Rat OA model chondrocytes by IL-1β pre-treatment were transfected with HA-PLCγ1 and HA-PLCγ1 Y783A vectors for 48 h, respectively, and PLCγ1, p-PLCγ1, Col 2, Aggrecan, and β-actin levels were detected by western blotting analysis using anti-PLCγ1, p-PLCγ1, Col 2, Aggrecan, and β-actin antibodies. (**D**) Rat OA model chondrocytes by IL-1β pre-treatment were transfected with myc-Akt and myc-Akt S473A for 48 h, respectively, and Akt, p-Akt, Col 2, Aggrecan, and β-actin levels were detected by western blotting using anti-Akt, p-Akt, Col2, Aggrecan, and β-actin antibodies. (**E**) Human OA Chondrocytes were transfected with HA-PLCγ1 and HA-PLCγ1 Y783A vectors for 48 h, respectively. Cell lysates were immunoprecipitated with PLCγ1 antibody, and then subjected to western blotting analysis with PLCγ1 and Akt antibodies. (**F**) Human OA Chondrocytes were transfected with myc-Akt and myc-Akt S473A for 48 h, respectively. Cell lysates were immunoprecipitated with Akt antibody, and then subjected to western blotting analysis with PLCγ1 and Akt antibodies. The same lysates were applied to ascertain the position and expression of PLCγ1 and Akt by western blotting analysis (Input). Data is representative of three independent experiments.

To understand the relationship between PLCγ1 and Akt, it was further detected the interaction between PLCγ1 and Akt in rat OA model chondrocytes by IL-1β (20 ng/ml) treatment. Given that both PLCγ1 and Akt, their phosphorylations are attributed to their regulatory effects on cell processes, the phosphorylation levels of PLCγ1 at Y783 site (p- PLCγ1) and Akt at S473 site (p-Akt) were measured with western blotting analysis. Figure 7A and 7B showed that TCN (10 μM) enhanced p-PLCγ1 level along with the decrease in p-Akt level and that U73122 (2 μM) enhanced p-Akt level along with the decrease in p-PLCγ1 level. Both of them were in a time-dependent manner. Figure 7C showed that the transfection with PLCγ1/Y783A mutant vector enhanced Col 2 and Aggrecan levels in rat OA model cells by IL-1β treatment, compared with PLCγ1-expressing vector group. Figure 7D showed that the transfection of Akt/S473A mutant vector reduced Col 2 and Aggrecan levels, compared with Akt-expressing vector group. These results indicated that the promoted effect of PLCγ1/Y783A mutant and the inhibitory effect of Akt/S473A mutant on matrix synthesis in rat OA model cells, similar to their pharmaceutical inhibitors in a rat OA model. Furthermore, a binding between PLCγ1 and Akt was noted in human OA chondrocytes using Co-immunoprecipitation assay (Figure 7E and 7F). Figure 7E also showed that PLCγ1/Y783A mutant reduced the binding between PLCγ1 and Akt, whereas Figure 7F indicated that Akt/S473A mutant enhanced the binding between them. Taken these data in chondrocytes *in vitro* together, there was also a reciprocal modulation between PLCγ1 and Akt and a binding between PLCγ1 and Akt, which was regulated by their phosphorylation levels.

## DISCUSSION

Given that targeting molecule therapy is thought to be an effective solution for OA therapy [2–4], and that the involvement of PLCγ1 and Akt in OA pathogenesis was reported by other authors’ and our previous studies [7–19], we sought to the possibility that PLCγ1 and Akt serve as potential molecular targets for OA therapy.

PLCγ1 has been detected at elevated level in human OA cartilage, implicated as a possible contributor to cartilage degeneration [13, 14]. The data in the study confirmed that different concentrations of PLCγ1 inhibitor could lead to a significant increase of cartilage thickness and decrease of OARSI scores (Figures 1 and 2). The elevated level of Col 2 and Aggrecan expression was measured in U73122 (PLCγ1 inhibitor)-treated groups (Figures 3 and 4). Similarly, PLCγ1/Y783A mutant also significantly delayed matrix degradation in human OA chondrocytes (Figure 7C). Thus, it is suggested that inhibition of PLCγ1, including its expression and phosphorylation, could protect chondrocytes against OA. In contrast, it has been addressed that activated Akt induced by extracellular factors or agents could promote cell proliferation and matrix synthesis to protect chondrocytes from OA degeneration [15–19]. Our data that no significant decrease of cartilage thickness and increase of OARSI scores were noted in TCN (Akt inhibitor)-treated rat OA model (Figures 1 and 2) demonstrates that the inhibition of Akt could not dramatically aggravate OA degeneration, implying the role of Akt may be inferior to that of PLCγ1 in OA pathogenesis. Perhaps, the intra-articular injection by Akt inhibitor interfered with many signal molecular cascades so as to have no obvious effect on OA pathogenesis. That the Akt/S473A mutant significantly suppressed matrix synthesis in human OA chondrocytes (Figure 7D) may be due to the discrimination between Akt/S473A mutant and inhibitor or intracellular and animal microenvironment. From the aforementioned data, we suggest that PLCγ1 inhibition may provide more attractive therapeutic target for OA therapy, than Akt activation.

In agreement with Wang Y *et al*. study that Akt binds to and phosphorylates PLCγ1 in response to epidermal growth factor [24], there was a binding between PLCγ1 and Akt in OA chondrocytes (Figure 7E and 7F). Moreover, we found that the binding between them was regulated by their phosphorylation levels. As Akt/S473A mutant suppressed matrix synthesis, the data that it led to the increased binding between PLCγ1 and Akt imply that the increased binding may aggravate matrix degradation. Similarly, PLCγ1/Y783A mutant that could promote matrix synthesis attenuated the binding between them, suggesting that the decreased binding conferred a benefit for matrix synthesis. Combined with the data that there was a mutual antagonism between PLCγ1 and Akt inhibitors in a rat OA model (Figures 5 and 6), the binding between PLCγ1 and Akt might be required for the mutual antagonism between them in OA chondrocytes, which will be studied in the future.

**Figure 8:**
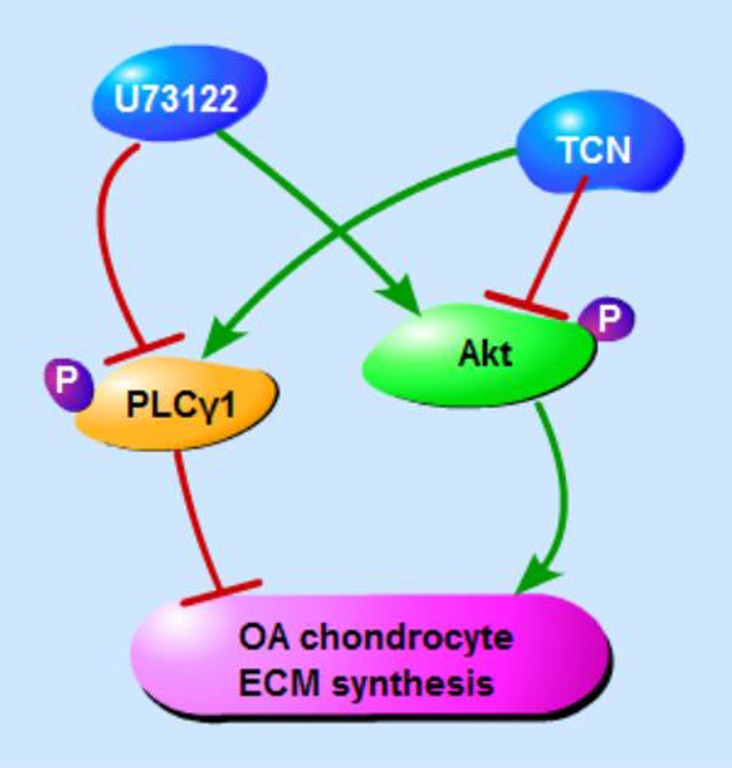
Schematic diagram of PLCγ1 and Akt regulating ECM synthesis of OA chondrocyte

In conclusions, PLCγ inhibition by its pharmaceutical inhibitor or PLCγ1/Y783A mutant could protect chondrocytes from OA degeneration in both a rat OA model and human OA chondrocytes, and that there is a mutual antagonism between PLCγ1 and Akt in a rat OA model (Figure 8). Furthermore, there was a binding between PLCγ1 and Akt, which was partially regulated by their phosphorylation levels in human OA chondrocytes. It is suggested that the inhibition of PLCγ1 may serve as a better solution for OA therapy, associated with the binding between PLCγ1 and Akt in OA chondrocytes.

## MATERIALS AND METHODS

### Reagents and antibodies

The antibodies against PLCγ1, p-PLCγ1 (Y783), Akt, p-Akt (S473), Aggrecan, Col 2, and β-actin were purchased from Cell Signalling Technology, Inc. (Beverly, MA, USA) and Santa Cruz Biotechnology (Santa Cruz, Freemont, CA, USA), respectively. Unless otherwise specified, inhibitors U73122 (U), Triciribine (TCN), and the rest of the reagents were purchased from Sigma-Aldrich (St. Louis, MO, USA).

### Establishment of a rat experimental model of OA

The protocol was approved by the Committee on the Ethics of Animal Experiments of the University of Xiamen (ID no.20140306). 9-week-old male Sprague–Dawley rats (250–300 g) were acclimatized to the laboratory environment for 1 week before the experiments and were randomly divided into two parts, 1 and 2 months. Each part included nine groups (*n* = 4), such as normal, OA, NS(intra-articular injection by 0.9% normal saline as control group), U73122 (U) (10 μg/kg, 100 μg/kg, 200 μg/kg), and Triciribine (TCN) (10 μg/kg, 100 μg/kg, 200 μg/kg). The rat experimental model of OA was induced with anterior cruciate ligament transection in combination with resection of medial menisci (ACLT + MMx) as previously described [18, 19]. The right knee joint of each rat was the experimental joint. After the 4th week post-surgery, different doses of inhibitors or NS were injected into the knee joints once every five days, and rats were not killed until 1 and 2 months after injection, respectively. This study was carried out in strict accordance with the recommendations in the Guide for the Care and Use of Laboratory Animals of the National Institutes of Health.

### Histopathological assay and evaluation for articular cartilage degeneration

The fresh samples were fixed in 4% paraformaldehyde for 48 h followed with decalcification in 10% EDTA-2Na for 3 weeks, and then paraffin-embedded for further routine histological preparation. Three micrometer-thick sections were deparaffnized in xylene and rehydrated in graded alcohols and distilled water prior to H.E. and Safranin O –Fast green stainings as previously described [18, 19]. The articular cartilage thickness of each femur condyle (from superficial zone to tidemark) was measured using Image-Pro Plus 6.0 software. According to OARSI scoring system established for grading OA changes [25], and semi-quantitative histopathological grading was performed using VS120-S6 (Olympus, Japan) by two different blinded pathologists, for a maximum possible score of 6. Grade 0 represents normal articular cartilage and increasing grade indicates a more biologically cartilage degeneration.

### Immunohistochemistry assay

According to the manufacturer's instructions (MAIXIN.BIO, Fuzhou, China), the sections were incubated overnight at 4°C with primary antibody: Col II (1: 1000), Aggrecan (1:800), PLCγ1 (1:200), and Akt (1:200) dilutions, respectively, prior to incubation with secondary antibodies. Diaminobenzidine (DAB) was then used to visualize the immunohistochemical reaction followed by being counterstained with haematoxylin. Finally, dark brown cells were considered to be positive. Photomicrographs were taken with OLYMPUS BX41 microscope equipped with a digital camera. The percentage of positive chondrocytes was measured and analyzed using Image-Pro Plus 6.0 Software and GraphPad Prism version 5, representing relative protein expression level [22, 23].

### Chondrocyte isolation and culture

Neonatal male Sprague–Dawley rats (within 24-72 hrs after birth) were killed after approval of the ethical Committee of Medical School, Xiamen University (ID No. 20110920). Articular cartilages of rat hint limbs were removed under sterile conditions and chondrocytes were then cultured as previously described [17, 18, 26]. F1 generation was treated with IL-1β (20 ng/ml) for 36 hr prior to the subsequent experiments.

In addition, after receiving all patient consent and in accordance with the hospital ethical guidelines approved by the Ethics Committee of Zhongshan Hospital, Xiamen University (ID No. 20100426), China, human OA cartilage was obtained from 79 patients (aged 42–74 years, 16 males and 63 females) with advanced OA who were undergoing total knee replacement surgery (Table 1). As described previously [19, 27], primary chondrocytes were cultured in Dulbecco's modified Eagle's medium (DMEM) containing 10% fetal bovine serum to 80% confluence and plated in 60-mm Petri dishes for the subsequent experiments. All of our clinical studies have been conducted according to the principles expressed in the Declaration of Helsinki.

**Table 1:** Information of OA patients with total knee replacement surgery

Age(Y)	Case	Sex		Duration of OA(Y)		^*^K.L. Image Criterion		Pro-treatment
		M	F	≤3	>3	III	IV	Arthroscopy
≤65	32	10	22	10	22	1	31	12
>65	47	6	41	2	45	4	43	19

### Western blotting and immunoprecipitation assay

Protein extracts were subjected to SDS-PAGE (6–10%, according to the molecular weight of protein) and transferred to PVDF membranes (Millipore, MA, USA) for western blotting analysis as described in previous studies [28, 29]. The signal was monitored using a chemiluminescent detection system according to the manufacturer's instructions (Pierce, Rockford, IL, USA).

As described previously [29, 30], protein extracts were lysed and 400 μg protein were mixed with 8 μl Protein A&G Sepharose (Sigma-Aldrich, Shanghai, China) and 8 μl anti-Akt or PLCγ antibody or immunoglobulin (IgG) control for 3 h at 4°C. The protein-antibody complexes that were recovered on the beads were subjected to Western blot analysis as above.

### Statistics analysis

All data were expressed as the means ± S.E.M. for three or five independent experiments for each group. The differences between the groups were examined for statistical significance using the unpaired *t*-test with GraphPad Prism version 5 (GraphPad Software, Inc., San Diego, CA. USA). A value of *P* < 0.05 was considered as being significant.

## Abbreviations

PLCγ: Phosphoinositide-specific phospholipase γ
PKB/Akt: protein kinase B
U: U73122
TCN: Triciribine
H.E.: Hematoxylin & Eosin
ACLT + MMx: anterior cruciate ligament transection in combination with resection of medial menisci
TIMP-1: tissue inhibitor of metalloproteinase
ADAMTS-5: a disintegrin and metalloproteinase with thrombospondin motifs 5
